# Variation in Capsidiol Sensitivity between *Phytophthora infestans* and *Phytophthora capsici* Is Consistent with Their Host Range

**DOI:** 10.1371/journal.pone.0107462

**Published:** 2014-09-09

**Authors:** Artemis Giannakopoulou, Sebastian Schornack, Tolga O. Bozkurt, Dave Haart, Dae-Kyun Ro, Juan A. Faraldos, Sophien Kamoun, Paul E. O’Maille

**Affiliations:** 1 The Sainsbury Laboratory, Norwich, United Kingdom; 2 Sainsbury Laboratory, Cambridge University, Cambridge, United Kingdom; 3 Imperial College, Faculty of Natural Sciences, Department of Life Sciences, London, United Kingdom; 4 Institute of Food Research, Food & Health Programme, Norwich, United Kingdom; 5 Department of Biological Sciences, University of Calgary, Calgary, Canada; 6 School of Chemistry, Cardiff University, Cardiff, United Kingdom; 7 John Innes Centre, Department of Metabolic Biology, Norwich, United Kingdom; Agriculture and Agri-Food Canada, Canada

## Abstract

Plants protect themselves against a variety of invading pathogenic organisms via sophisticated defence mechanisms. These responses include deployment of specialized antimicrobial compounds, such as phytoalexins, that rapidly accumulate at pathogen infection sites. However, the extent to which these compounds contribute to species-level resistance and their spectrum of action remain poorly understood. Capsidiol, a defense related phytoalexin, is produced by several solanaceous plants including pepper and tobacco during microbial attack. Interestingly, capsidiol differentially affects growth and germination of the oomycete pathogens *Phytophthora infestans* and *Phytophthora capsici*, although the underlying molecular mechanisms remain unknown. In this study we revisited the differential effect of capsidiol on *P. infestans* and *P. capsici,* using highly pure capsidiol preparations obtained from yeast engineered to express the capsidiol biosynthetic pathway. Taking advantage of transgenic *Phytophthora* strains expressing fluorescent markers, we developed a fluorescence-based method to determine the differential effect of capsidiol on *Phytophtora* growth. Using these assays, we confirm major differences in capsidiol sensitivity between *P. infestans* and *P. capsici* and demonstrate that capsidiol alters the growth behaviour of both *Phytophthora* species. Finally, we report intraspecific variation within *P. infestans* isolates towards capsidiol tolerance pointing to an arms race between the plant and the pathogens in deployment of defence related phytoalexins.

## Introduction

Plants are exposed to a variety of disease causing organisms, including viruses, bacteria, fungi, oomycetes, nematodes, insects, and parasitic plants [1], [2]. Yet, one concept in plant pathology is that in general plants are resistant to most pathogens. Plants have evolved a defense system that enables them to produce compounds that affect microbes in various ways. Some of these compounds are broad spectrum, whereas others are not. Among such defence compounds are phytoalexins, which are antimicrobial specialized metabolites that are induced under stress conditions or upon infection by a pathogen [3]–[5]. The spectrum of action of phytoalexins remains poorly understood and, surprisingly, their contribution to species-level (also known as nonhost) resistance is not always fully appreciated.

One well-studied phytoalexin is capsidiol, which is produced by the solanaceous plants *Capsicum annuum* (pepper) or *Nicotiana tabacum* (tobacco) after infection by pathogens such as the oomycete *Phytophthora capsici* [6], [7]. Remarkably, capsidiol affects diverse pathogens such as fungi and oomycetes. [3]–[6], [8]–[11]. Capsidiol is a bicyclic sesquiterpenoid compound and member of the isoprenoid class of phytoalexins. Like all sesquiterpenes, capsidiol derives from a common substrate farnesyl diphosphate (FPP) [12]. Two key enzymes are responsible for the biosynthesis of capsidiol. 5-*epi*-aristolochene synthase (EAS) catalyzes the cyclization of FPP to the intermediate 5-*epi*-aristolochene, then 5-*epi*-aristolochene dihydroxylase (EAH) mediates the two hydroxylation steps at positions C-1 and C-3 of 5-*epi*-aristolochene to yield capsidiol [13]. The dihydroxylase works in parallel with a cytochrome P450 reductase (CPR; NADPH-ferrihemoprotein reductase), which transfers electron equivalents for EAH reactions.

The oomycete genus *Phytophthora* includes some of the most destructive plant pathogens [14]. Several *Phytophthora* spp. infect solanaceous plants, including important crops like potato, tomato and pepper. Two of the most notorious species are the potato and tomato late blight pathogen *Phytophthora infestans* and the vegetable pathogen *P. capsici*. Both *P. infestans* and *P. capsici* have emerged as model systems to study oomycete pathogens and they have been extensively studied at the genomic level [15]–[19]. These species follow a hemibiotrophic life style and adopt two separate phases during infection. In the early stage of infection, both pathogens need living host cells. This biotrophic phase is followed by extensive necrosis of host tissue (necrotrophic phase) [20]. The host range of *P. infestans* is limited to solanaceous plants, particularly potato and tomato, whereas *P. capsici* affects a wide range of hosts in the Cucurbitaceae, Fabaceae, and Solanaceae families [14]. Although these two *Phytophthora* species share a common host in tomato, *P. infestans* cannot infect several host plants of *P. capsici*, notably pepper. Nonhost resistance to *P. infestans* is associated with a plant localized cell death response also known as the hypersensitive response (HR) [21].

The molecular basis of host-specificity of *Phytophthora* species, such as *P. infestans* and *P. capsici* is unknown. Although disease resistance genes that operate at the nonhost level are likely to be implicated [22], early work has also suggested a role for phytoalexins. For example, in the 1970s, several studies have shown that capsidiol has differential activity against *P. infestans* and *P. capsici* [4], [5]. Jones et al. showed that *P. infestans* is more sensitive (∼10 fold) to capsidiol than *P. capsici*, both in spore germination and growth assays [4]. Jones et al. also showed that, below a certain threshold, capsidiol has a reversible effect on both *Phytophthora* species [4]. This level of capsidiol is only reached in vivo in pepper varieties that are resistant to *P. capsici*, which led the authors to suggest sensitivity to capsidiol and differential accumulation of this phytoalexin might determine host susceptibility [23]. Apart from these pioneering studies that date back to the 1970s, only few publications have examined the role of capsidiol in *Phytophthora* pathosystems except to use it as a marker for defense [6], [8], [10], [24]. Nonetheless, Shibata et al. showed that silencing of *NbEAS* and *NbEAH*, two ethylene-regulated genes for capsidiol biosynthesis, negatively impact the resistance of *Nicotiana benthamiana* against *P. infestans* suggesting a positive role of capsidiol in this interaction [25], [26].

In this study, we revisited the effect of capsidiol on *P. infestans* and *P. capsici*, and the variation in sensitivity to this phytoalexin. Compared to the earlier studies [4], [11], [23], [27] we used highly pure preparations obtained from yeast engineered to express the capsidiol biosynthetic pathway [28]. We also assayed the effect of capsidiol on both mycelial growth and zoospores, using a novel fluorescence-based assay taking advantage of transgenic *Phytophthora* strains expressing fluorescent markers for biomass quantification. We confirmed major differences in capsidiol sensitivity between *P. infestans* and *P. capsici*. We also showed that capsidiol alters the growth behaviour of both *Phytophthora* species. Finally, we monitored the intraspecific variation within *P. infestans* isolates to capsidiol.

## Results

### *P. infestans* is more sensitive to capsidiol than *P. capsici*

**Figure 1:**
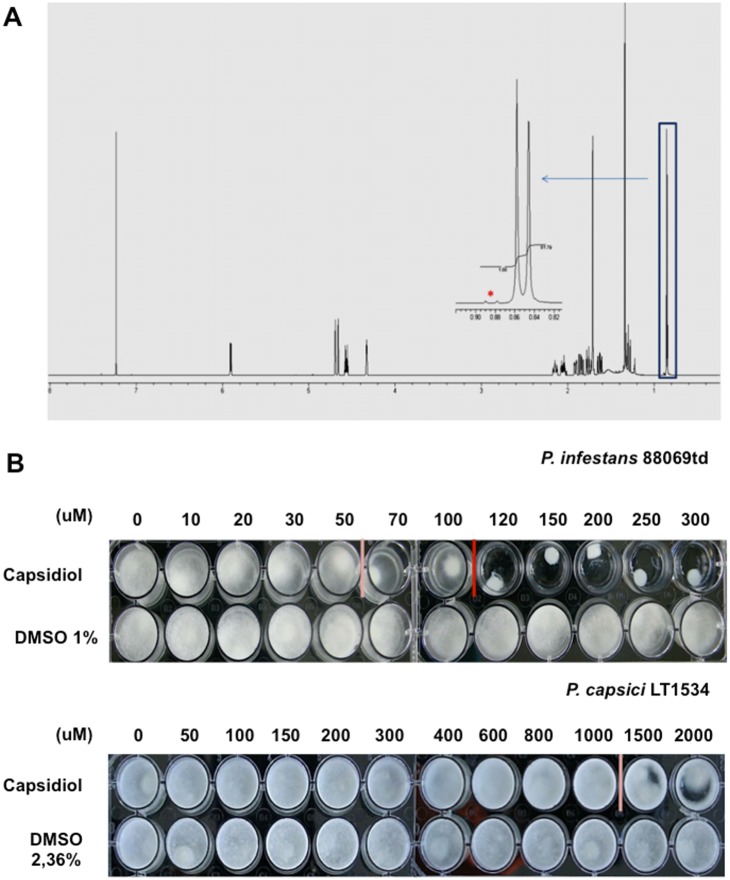
*P. infestans* is more sensitive to capsidiol than *P. capsici*. (A) Verification of capsidiol purity as tested by NMR spectroscopy (Nuclear Magnetic Resonance Spectroscopy). ^1^H NMR (CDCl_3,_ 600 MHz) spectrum of capsidiol. NMR integrations of the diagnostic methyl doublet at δ_H_ 0.88 ppm (expansion) reveal a purity of greater than 98.8%. (*) Represents the impurity. (B) Growth inhibition assay of *P. infestans* and *P. capsici* after 10 days of exposure of mycelial plugs to capsidiol. Pink bar delineates the lowest concentration with an inhibitory effect and the red bar the concentration after which there is no longer growth. This experiment was performed 4 times.

To examine the effect of capsidiol on *Phytophthora* spp., we conducted inhibition assays using mycelial plugs of 2 to 3 week-old plates of *P. infestans* and *P. capsici,* which were placed in sterilised 26-well plates (Greiner Bio-one) in a rich medium, supplemented with varying concentrations of capsidiol. In our experiments, we used a metabolically engineered yeast system [28] to produce high purity capsidiol as shown by Nuclear Magnetic Resonance (NMR) Spectroscopy (Fig. 1A). Mycelial growth was assessed by visual inspection after 10 days of incubation of agar-grown mycelial plugs in capsidiol- or control-containing liquid medium at 20°C in the dark for *P. infestans* and 25°C and illumination for *P. capsici*. We observed reduced *P. infestans* growth at capsidiol concentrations of 50 µM or above and no growth was observed at concentrations of 120 µM and higher. *P. capsici* growth was affected at capsidiol concentrations of 1.5 mM or higher, but was not fully inhibited in any of the tested capsidiol concentrations (Fig. 1B). Since capsidiol stock solution was dissolved in DMSO, we also tested whether DMSO affects mycelial growth of *Phytophthora*. We found, that DMSO did not affect *Phytophthora* growth at concentrations below 2.5% (v/v), which is equivalent to the highest relative DMSO concentration that was used during the experiment. In summary, our results confirm earlier indications that *P. capsici* displays a higher degree of resistance to capsidiol than *P. infestans*. However, in our hands complete growth inhibition of *P. infestans* was achieved with 120 µM capsidiol, a value 2 times less than previously reported [4].

### Capsidiol arrest of *P. infestans* growth is reversible

**Figure 2:**
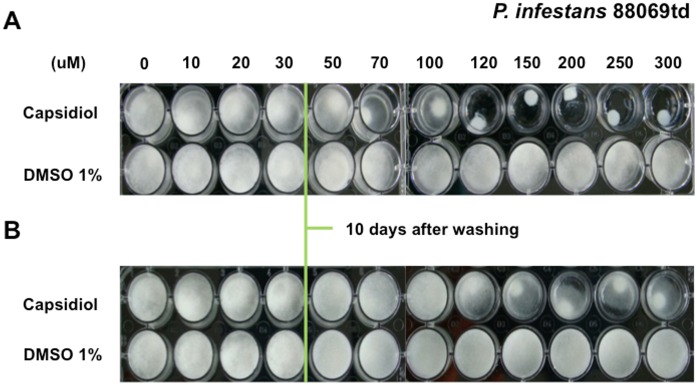
Capsidiol inhibits *P. infestans* growth reversibly. (A) Growth inhibition assay of *P. infestans* after 10 days of exposure of mycelial plugs to capsidiol. (B) Restoration of growth after washing treatment. Green line indicates the point after which the washing treatment was applied. The experiment was performed 3 times. Picture was taken 10 days after the washing and 20 days after initial exposure to capsidiol.

It has been reported [4], [23] that growth inhibiting effects of capsidiol are reversible at concentrations below 5 mM, while higher capsidiol concentrations are considered to be fungitoxic [23]. Following our plug inhibition assays, we studied the reversibility of capsidiol growth inhibition using the previously established *P. infestans* microtitre plate assay. The capsidiol-containing medium was removed from the wells and the mycelia were washed 3 times with deionised water, after which fresh liquid culture medium (Plich) was added. Growth restoration was observed 24 hours after washing and 10 days later the extent of mycelial growth was similar to the control that was grown without any capsidiol (Fig. 2B). This finding is consistent with reports that low capsidiol concentrations reversibly inhibit *Phytophthora* growth [4].

### Quantitative evaluation of differential growth inhibition of *P. infestans* and *P. capsici* by capsidiol

**Figure 3:**
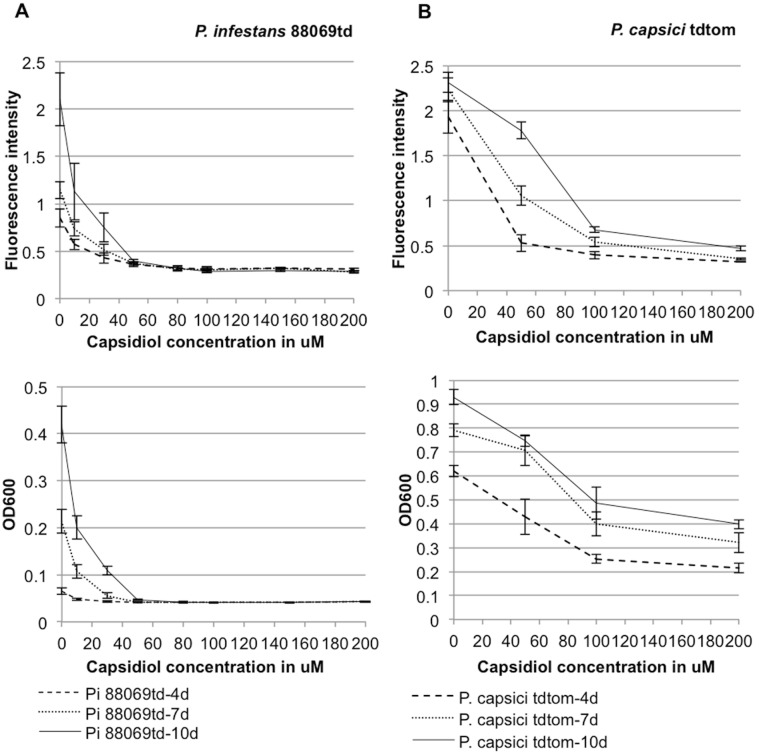
Capsidiol is not affecting *P. capsici* growth as severely as it does *P. infestans*. (A) Dose response curves of *P. infestans* 88069td calculated at 4, 7 and 10 days for both Fluorescence intensity and OD600. (B) Dose response curves of *P. capsici* tdtom calculated at 4, 7 and 10 days for both Fluorescence intensity and OD600.

In order to quantify the effect of capsidiol on the growth of *Phytophthora* strains, we developed and applied an inhibition assay with zoospore suspension solutions and measured the amount of growing mycelia using either optical density or emitted fluorescence of transgenic *Phytophthora* strains. For this experiment we used *P. infestans* 88069td, *P. infestans* 88069 [29], *P. capsici* LT1534 tdtom and *P. capsici* LT1534 [16] strains (td and tdtom strains are transgenic strains expressing the red fluorescent marker tandem dimer RFP, known as tdTomato). Zoospores were harvested from *Phytophthora* plates and incubated with various concentrations of capsidiol in Plich medium in microtitre plates. The plates were scanned at 1 to 3 day intervals for OD600 (Optical Density at 600 nm) and red fluorescence intensity. Dose response curves were obtained by measuring both red fluorescence intensity and OD600 at increasing capsidiol concentrations to directly compare the difference in sensitivity between *P. infestans* and *P. capsici* (Fig. 3).

**Figure 4:**
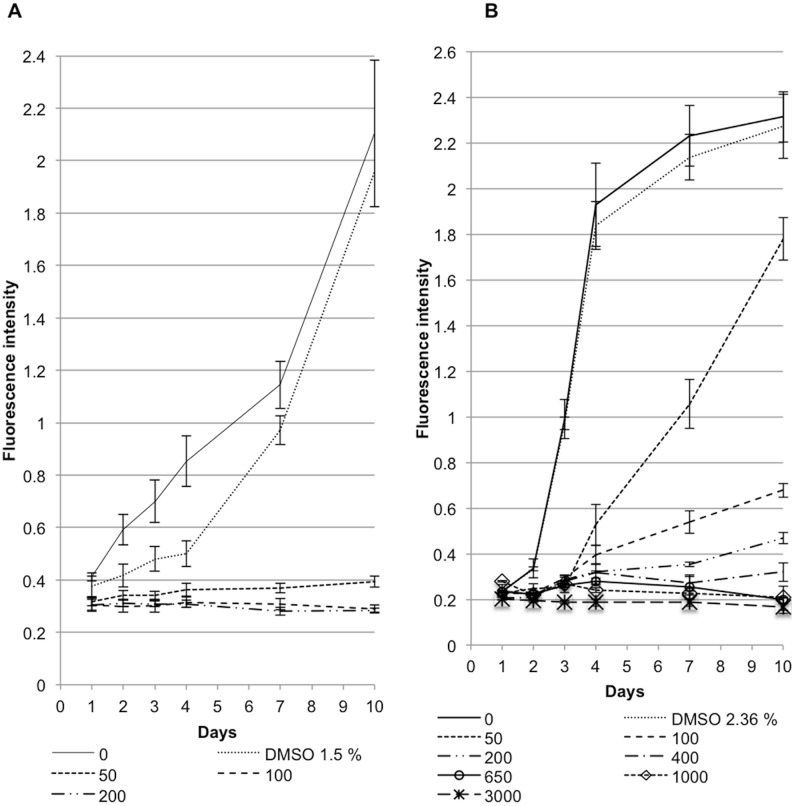
Scatter plots correlating fluorescence intensity and capsidiol concentration. The plots illustrate fluorescence intensity of *P. infestans* 88069td (A) and *P. capsici* tdtom (B) strains over time for a maximum of 10 days. The experiment was performed 3 times.

Results from measurements of red fluorescence intensity under capsidiol treatment, revealed statistically significant difference between *P. infestans* and *P. capsici* (Fig. 4). All concentrations of capsidiol above 50 µM dramatically affected the ability of *P. infestans* 88069td to emit red fluorescence. The given values were at a range of 0.3 red fluorescent units (RFU) after 10 days, close to the value obtained with the non-fluorescent 88069 strain (Fig. 4A and S1). On the contrary, *P. capsici* tdtom retained its ability to emit red fluorescence up to a concentration of 650 µM of capsidiol, after which RFU levels dropped down to the non-fluorescent *P. capsici* strain values (Fig. 4B and S1).

**Figure 5:**
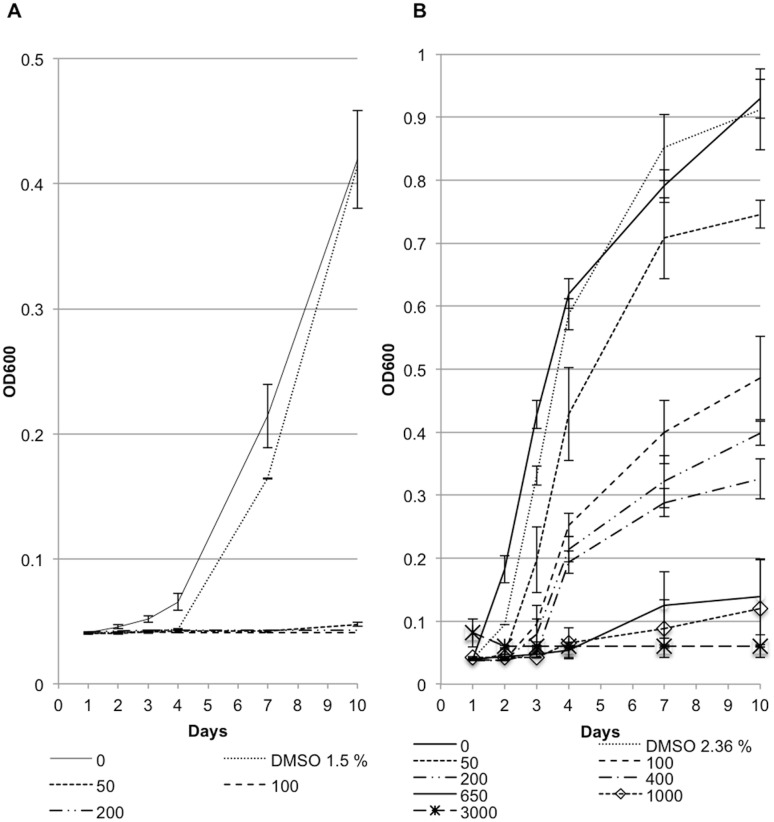
Scatter plots correlating 0D600 and capsidiol concentration. The plots illustrate growth of *P. infestans* 88069td (A) and *P. capsici* tdtom (B) strains over time for a maximum of 10 days. The experiment was performed 3 times.

The observed growth differences could also be reported using OD600 measurements (Fig. 5). Capsidiol concentrations of 50 µM or greater were detrimental for *P. infestans* growth, after which it was suppressed at OD600 levels lower than the control strain (Fig. 5A and S1). *P. capsici* growth was severely affected by capsidiol concentrations of 650 µM and higher, where the OD600 values were close to the ones of the control strain (Fig. 5B).

These results corroborate the findings that *P. capsici* is more resistant to capsidiol than *P. infestans* and revealed that the difference in sensitivity is almost 13 fold. DMSO did not affect the red fluorescence intensity or OD600 of any of the *Phytophthora* strains at concentrations below 2.36% (v/v), a value equivalent with the maximum capsidiol solution that was used during the experiment.

### Capsidiol alters *P. infestans* and *P. capsici* mycelial growth

**Figure 6:**
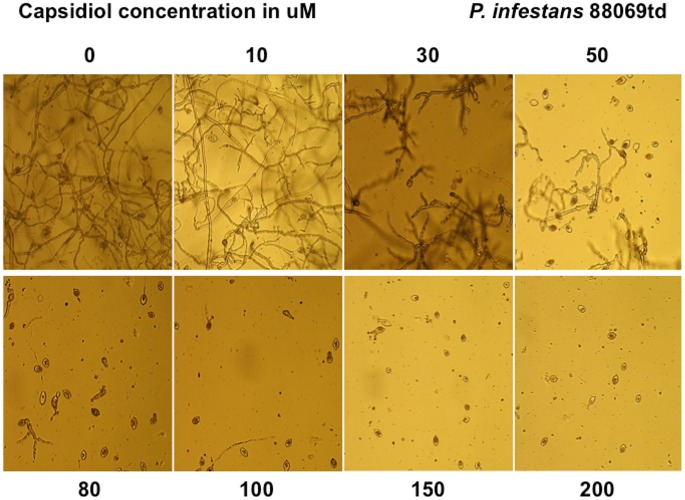
Growth behaviour of *P. infestans* 88069td, after 10 days of exposure to different capsidiol concentrations. The experiment was performed 3 times.

**Figure 7:**
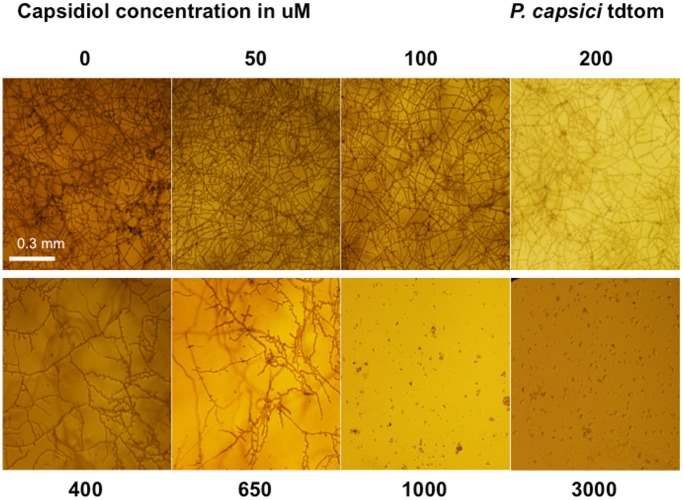
Growth behaviour of *P. capsici* tdtom, after 10 days of exposure to different capsidiol concentrations. The experiment was performed 3 times.

In order to study the effects of capsidiol-mediated inhibition of mycelial growth of *Phytophthora* we microscopically monitored the hyphal morphology during a capsidiol time course treatment at 2–4 day intervals in microtitre plates. When monitoring hyphal growth of *P. infestans* 88069td (Fig. 6) and *P. capsici* tdtom (Fig. 7) we observed that capsidiol alters *P. infestans* growth more severely and is effective at concentrations of 10 µM, whereas *P. capsici* remains unaffected. Stunted branching of *P. capsici* tdtom mycelia was observed at capsidiol concentrations of 400 µM. DMSO did not have any effect on growth for either *P. infestans* 88069td or *P. capsici* tdtom (Fig. S2). These results are in agreement with the limiting capsidiol concentrations obtained in zoospore inhibition assays for both species.

### Variation in sensitivity to capsidiol among *P. infestans* isolates

**Figure 8:**
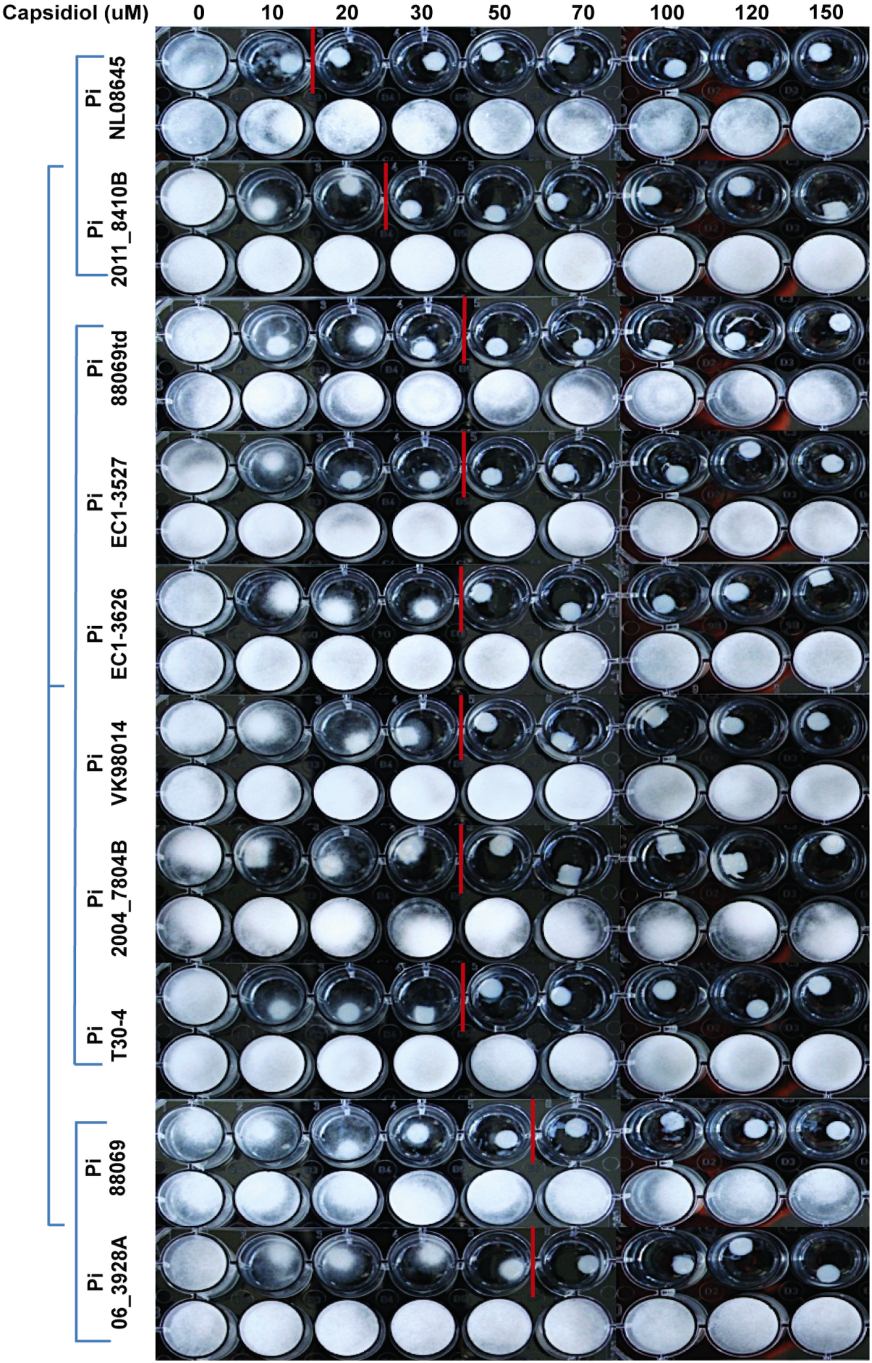
Different *P. infestans* isolates have different sensitivity to capsidiol. Isolates are clustered according to their sensitivity, starting from the most sensitive to the least. Top row of wells of each isolate represents capsidiol treatment (capsidiol was dissolved in DMSO/Plich media) in µM and the lower row represents treatment with 1% (v/v) DMSO/Plich (negative control). The experiment was performed 3 times.

To identify differences in sensitivity towards capsidiol between various *P. infestans* isolates, we conducted an experiment exposing mycelial plugs to various concentrations of capsidiol, as described above. For this experiment we used the following *P. infestans* isolates: 88069 [30], 88069td [31], [32], T30-4 [15], 06_3928A [18], VK98014 [33], EC1-3527, EC1-3626, 2004_7804B [18], 2011_8410B [18] and NL08645 [15] (Table 1). We found that only one isolate, 06_3928A, displayed a similar level of resistance to capsidiol as our reference isolate, 88069, whereas the other isolates were more sensitive with isolate NL08645 being the most sensitive to capsidiol (Fig. 8). DMSO did not have any effect on *Phytophthora* growth in the concentrations used to dilute capsidiol. Our results support strain-specific variation of *P. infestans* isolates to capsidiol growth inhibition, the genetic basis of which remains to be studied.

**Table 1:** Provenance of *Phytophthora* samples.

Isolate ID	Country of origin	Collection year	Host species	Reference
88069	The Netherlands	1988	*Solanum lycopersicum*	Van West et al. (1998)
88069td				Whisson SC et al. (2007)
T30–4				Haas et al. (2009)
06_3928A	United Kingdom	2006	*Solanum tuberosum*	Cooke, Cano et al. (2012)
VK98014	The Netherlands	1998	*Solanum tuberosum*	G. J. T. Kessel et al. (2012)
EC13527	Ecuador	2002	*Solanum andreanum*	World Oomycete Genetic Resource Collection at UC Riverside, CA
EC13626	Ecuador	2003	*Solanum tuberosum*	World Oomycete Genetic Resource Collection at UC Riverside, CA
2004_7804B	Scotland	2004	*Solanum tuberosum*	Cooke, Cano et. al, (2012)
2011_8410B	United Kingdom	2011	*Solanum tuberosum*	Cooke, Cano et. al, (2012)
NL08645	The Netherlands	2008	*Solanum venturii*	G. J. T. Kessel et al. (2012)

## Discussion

In this work, we developed new assays to examine the effect of the phytoalexin capsidiol on two *Phytophthora* species with differing host ranges. Our results are overall consistent with a 1975 report [4] that *P. infestans* is more sensitive to capsidiol than *P. capsici* using highly pure preparations of capsidiol. A major (>10-fold) differential effect of capsidiol between species was noted using both mycelial and zoospore assays. Considering that this phytoalexin is produced by pepper but not potato, our findings are consistent with the hypothesis that capsidiol contributes to nonhost resistance of pepper to *P. infestans*.

Previous studies used capsidiol purified from pepper fruits or tobacco cell cultures [4], [11], [23], [27]. We used a recently developed method to produce highly pure capsidiol synthesized in yeast [28]. This reduced the likelihood that contaminating phytochemicals may have affected the experiments. It allowed us to directly test the effect of capsidiol on *Phytophthora* species and helped us to more accurately estimate the inhibitory doses of capsidiol on *Phytophthora* growth. Furthermore, we took advantage of fluorescently labelled *Phytophthora* strains to measure biomass and growth. Although our findings are consistent with the earlier studies, we could more accurately estimate the difference in sensitivity. We found that 120 µM of capsidiol completely inhibited growth of *P. infestans* both in mycelial and zoospore assays, whereas Jones et al. concluded that this effect started at 200 µM of capsidiol [4]. Furthermore, in our mycelial plug assays *P. capsici* was not completely inhibited even at a concentration of 2 mM capsidiol, whereas according to Jones et al. 1.5 mM is a completely inhibitory concentration [4]. However, these differences are probably due to the assays used. Our zoospore assays were more consistent with the results of Jones et al. who concluded that capsidiol has a fungistatic effect at 3.75 mM and is fungitoxic at concentrations that exceed 5 mM [4], [23]. We also found that the difference in sensitivity to capsidiol between the two *Phytophthora* species is approximately 13 fold, which is in agreement with earlier studies that showed *P. capsici* to be at least 10 times more resistant to capsidiol than *P. infestans* [4]. Finally we extended our studies and showed that the level of sensitivity between different *P. infestans* isolates varies, providing a basis for studying the underlying genetic variation.

Earlier studies have showed that similar to capsidiol, other phytoalexins show a differential toxicity to phytopathogenic fungi. Hargreaves et al. [34] showed that the major phytoalexins from *Vicia faba* including isoflavoinoid medicarpin and wyerone acid had a greater impact on germ tubes produced by the necrotrophic fungus *Botrytis cinerea*, than *B. fabae*. They further highlighted a differential toxicity in wyerone derivatives than medicarpin [34]. Also, another study on the effect of the phytoalexins pisatin and maackiain from garden pea and red clover, respectively, against 19 fungal species revealed that nonhost phytoalexins have a greater effect inhibiting growth of the pathogens tested than the phytoalexins naturally occurring in the host [35]. These studies point out that differential activity of phytoalexins is a common phenomenon, and highlight the importance of understanding how different pathogens have evolved to cope with them.

What could be the nature of the differential effect of capsidiol on the two *Phytophthora* species? Ward and Stoessl [36] argued that *P. capsici* detoxification of capsidiol is unlikely and instead proposed that *P. capsici* does not induce high enough levels of capsidiol during infection of its host plant pepper [4], [36], [37]. Detoxification would probably involve oxidation of capsidiol to a less fungitoxic ketone, capsenone, as noted in *in* *vitro* assays with the fungi *Botryris cinerea* and *Fusarium spp.* [3], [36]. Importantly, capsenone was not detected in pepper tissue infected with *P. capsici* indicating that the pathogen may evade the phytoalexin by limiting its induction [36]. Alternatively, ATP-binding cassette (ABC) transporters may be involved as an efflux pump. Coleman et al. showed that the rot causing ascomycete *Nectria haematococca* can overcome the effect of the pea phytoalexin pisatin using a specific ABC transporter, NhABC1, that enhances the fungus tolerance to the phytoalexin [38]. Since there is no evidence that *P. capsici* can detoxify capsidiol [36], [37], *P. capsici* may rely on ABC transporters to cope with capsidiol. A more recent study on the role of ABC transporters in fungicide sensitivity in *P. infestans* failed to show correlation between up-regulation of ABC transporter genes in strains that are less sensitive to fungicides [39]. Whether inter- or intra-specific variation in expression of ABC transporter genes explains differences in capsidiol sensitivity in *Phytophthora* remains to be determined.

A genetic difference in the target of capsidiol could underpin the difference in sensitivity between the *P. capsici* and *P. infestans*. De Marino et al. [40] showed that capsidiol has a bacteriostatic effect against the human gastritis pathogen *Helicobacter pylori in* *vitro* but the mode of action remains unknown. It would be interesting to identify the molecules that are targeted by capsidiol in *Phytophthora*. Given that the genome sequences of *P. capsici* and *P. infestans* are available [15], [41], [42], a promising approach would be to determine transcriptome dynamics in response to capsidiol. From an evolutionary perspective, it would be of great interest to examine the response of *Phytophthora* to other sesquiterpenes that emerged during the functional divergence of terpene synthases in solanaceous plants [43].

The differences in capsidiol sensitivity observed among various *P. infestans* isolates reflect the remarkable level of diversity noted in this highly adaptable plant pathogen species [18], [21]. This variation is similar to what has been noted for sensitivity to fungicides in *P. infestans* and other oomycetes [44]–[46]. In some cases the genetic basis of chemical sensitivity has been identified. Randall et al. determined that sequence polymorphisms in the large subunit of RNA polymerase I (RPA190) contributes to *P. infestans* insensitivity to the oomycete-specific control chemical Mefenoxam [44]. Also, Blum et al. demonstrated that for two oomycetes, the causal agent of downy mildew in grape, *Plasmopara viticola* and *P. infestans*, an amino acid change in a protein known to be involved in cellulose biosynthesis (PiCESA3 and PvCESA3 in the two pathogens respectively) confers insensitivity to Mandipropamide [45], [46].

We observed that sensitivity to capsidiol ranged ∼5 fold in the *P. infestans* isolates tested. Is there a biological significance for these differences? Although potato does not produce capsidiol, it is possible that *P. infestans* has evolved mechanisms to tolerate other terpenoids produced by potato, which might contribute to host immunity. Indeed, potato is known to accumulate rishitin, another bicyclic sesquiterpene phytoalexin that is related to capsidiol [22]. In the future, it would be interesting to examine whether there is any correlation between aggressiveness and tolerance to capsidiol among various *P. infestans* isolates.

Finally, our work points to a biotechnological approach to engineer resistance to *P. infestans*. Genetic manipulation of capsidiol production in *Nicotiana benthamiana*, a *P. infestans* host plant that produces capsidiol, has already indicated that this phytoalexin contributes to disease resistance [25], [26]. Interestingly, *P. capsici* is markedly more aggressive pathogen of *N. benthamiana* than *P. infestans* [29], possibly because it can tolerate the capsidiol produced by this plant. Ultimately, capsidiol biosynthetic genes could be transferred from pepper or tobacco to potato and tomato as a potential strategy for disease resistance against *P. infestans*.

## Materials and Methods

### Yeast growth

The yeast strain EPY300 was engineered to express the capsidiol biosynthetic pathway [28] and was used to produce capsidiol by fermentation. In brief a starter culture (ca. 20 ml) was prepared and inoculated into a 5 L-bioreactor containing rich media to full capacity. The media consisted in 1% Bacto yeast extract, 2% Bacto peptone (BD Biosciences, Oxford, UK), 1.8% galactose, 0.2% glucose, 150 mg/L methionine and 80 mg/L adenine hemisulphate (Sigma Aldrich Co Lt, Dorset, UK). The bioreactor was set to 30°C, with constant stirring (180 rmp) and aeration at 4 L/min. After 96 hours both stirring and aeration were stopped, and the temperature was reduced to 5°C. Once yeast cells had settled (24–48 h), the media containing the yeast-produced capsidiol was decanted for extraction.

### Capsidiol extraction and purification

Capsidiol was isolated by dichloromethane extractions of the media. The combined extracts (eg 5 L total volume) were dried, filtered and evaporated to dryness using a rotary evaporator. The crude extract (ca. 1,500 mg) was re-dissolved in a minimum volume of 1∶1 hexane/ethyl acetate (EA) and applied to a glass sinter funnel 40 mm×40 mm containing silica gel (previously equilibrated with hexane), and connected to an on-house pump. Purification of capsidiol was accomplished by vacuum filtration using a gradient (0–66%) of ethyl acetate/hexane. Each fraction (25 mL) was assessed for capsidiol (R_f_ = 0. 363) content by analytical TLC (Merck silica gel 60 (F_254_) 7×7 cm aluminium-coated plates), using 66% ethyl acetate/hexane as the developing solvent and visualization with CAM solution (cerium ammonium molybdate). Fractions judged to contain exclusively capsidiol were combined together and evaporated under reduced pressure. Further (and final) purification of this material was effected by preparative TLC (Merck silica gel 60 (F_254_) 20×20 cm glass-coated plates) previously sprayed with a 0.5% berberine chloride ethanolic solution (non-destructive visualization of capsidiol by UV at 365 nm). Briefly, the silica gel TLC plates were divided horizontally in two halves by removing a thing line of silica coating and the sample (containing around 25 mg of product) was placed in a continuous line 1 cm above the bottom of the plate. After loading, the band was ‘focused/concentrated’ twice by standing the plate in a tank containing pure EA until the solvent front reached 2 cm. After air-drying, the plate was finally developed using 66% ethyl acetate/hexane. The band corresponding to capsidiol (R_f_ = 0.363) after visualization by UV light (365 nm) was marked and scraped off avoiding the very bottom of the band, which was shown to contain an as yet unidentified more polar terpene compound. The silica gel scrapings were loaded into a pipette-column and washed using ethyl acetate.

Typically we found that 5 L fermentation yielded around 1,500 mg of crude extract, which is reduced to about 700 mg after silica column, to produce 300–400 mg of essentially pure capsidiol (>97% by 1H-NMR) after preparative TLC.

### Identification of capsidiol

The unambiguous identification and purity estimation of the yeast-produced capsidiol was carried out by NMR spectroscopy and combined liquid chromatography-mass spectroscopy (LC/MS) following the method of Literakova et al. [27] with slight modifications. In brief, we used an isocratic 75% methanol: water solvent mixture, in a C8 reverse column (Agilent 1100 MSD) with negative mode TIC and SIM at m/z 201, 219, 259.

### *Phytophthora* cultivation

*Phytophthora* strains were grown on rye sucrose agar as previously described [47] at 20°C in the dark (*P. infestans*) or on V8 vegetable juice agar [7] plates (*P. capsici*) at 25°C and illumination. For the plug inhibition assays, 5 mm diameter plugs were taken from 2–3 week-old *Phytophthora* plates and placed in the wells of a 24-well plate, previously filled with 1 ml of Plich medium [2.4 gr sucrose, 0.27 gr asparagine, 0.15 g KH_2_P0_4_, 0.10 gr MgS0_4_ 7H_2_O, 10 mg cholesterol, 10 mg ascorbic acid, 2 mg thiamine HCl, 4.4 mg ZnSO_4_ 7H_2_O, 1 mg FeSO_4_ 7H_2_O, 0.07 mg MnCl_2_ 4H_2_O and 20 g agar (Difco) dissolved in 1 L deionized water [48]. For the zoospores inhibition assays, spores were harvested as previously described [47], [49] and diluted to 50,000 spores/ml. Droplets of 10 µl were added to each well of a 96-well plate, previously filled with 250 µl of Plich medium. Plates were kept at 20°C in the dark and 25°C and illumination for *P. infestans* and *P. capsici* respectively. Washes were applied to the plates containing the *Phytophthora* plugs by carefully removing the Plich media from the wells, adding distilled water, expose for 1 to 2 minutes and remove. This step was repeated at least 2 times. Finally 1 ml of fresh Plich media was added and plates were kept at 20°C in the dark (*P. infestans*) and 25°C and illumination for *P. capsici*.

### Spectroscopic growth assays

For the zoospore inhibition assays, 10 µl zoospore solution per well was distributed into 96-well microtitre plates (Greiner bio-one), covered with a plastic lid (Greiner bio-one), sealed with Parafilm (Pechiney Plastic Packaging Company) and incubated at 25°C in the dark for *P. infestans* and at 25°C and illumination for *P. capsici*, over 10 days. At regular intervals, mycelial growth was monitored using a Varioscan Flash Multimode Reader (Thermo Scientific) by measuring light absorption at OD600 as well as emission of red fluorescence (excitation at 360 nm, emission at 465 nm).

### Light microscopy

Mycelia grown in 96-well microtitre plates were imaged using a Zeiss Axiovert 25 microscope in transmission light mode with 10x magnification. Pictures were taken using a Cannon E0S-D30 camera.

## Supporting Information

Figure S1 **Fluorescence intensity of the non-fluorescent stains** ***P. infestans*** **88069 and** ***P. capsici*** **LT1534.** These strains were used as controls to verify that the signal in the fluorescent strains corresponds to fluorescence.
(TIF)

Figure S2 **Growth behaviour of** ***P. infestans*** **88069 and** ***P. capsici*** **LT1534 after exposure to DMSO.** Both Phytophthora strains were exposed to 1.5% and 2.36% (v/v) DMSO/Plich for 10 days. DMSO levels correspond to the maximum capsidiol solution that was used in each experiment. The experiment was performed 3 times.
(TIF)
