# Supplementary figures and images for: Variation in Capsidiol Sensitivity between Phytophthora infestans and Phytophthora capsici Is Consistent with Their Host Range
Source: PLoS One. 2014 Sep 9;9(9):e107462. doi: 10.1371/journal.pone.0107462 (PMC4159330; doi:10.1371/journal.pone.0107462)

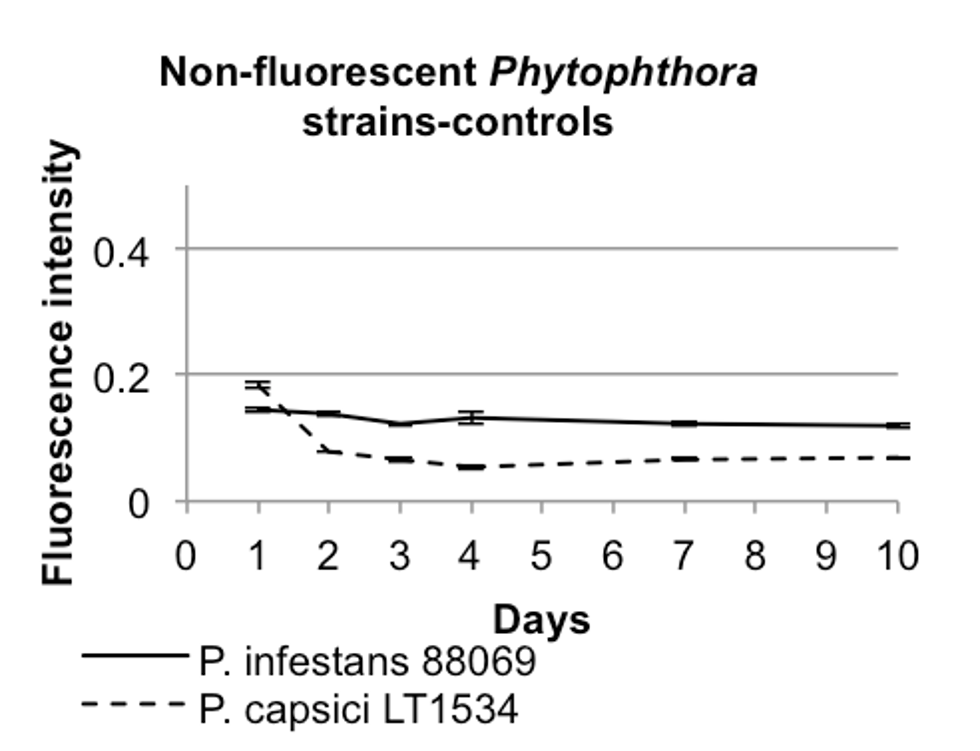

Supplement: Figure S1 — Fluorescence intensity of the non-fluorescent stains P. infestans 88069 and P. capsici LT1534. These strains were used as controls to verify that the signal in the fluorescent strains corresponds to fluorescence. (TIF) [file pone.0107462.s001.tif]

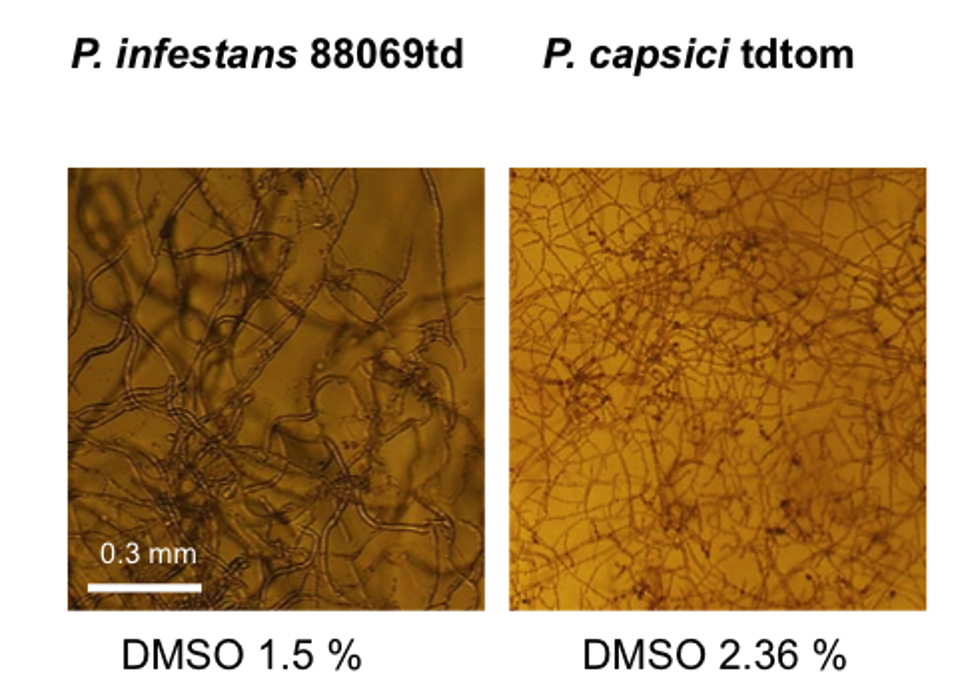

Supplement: Figure S2 — Growth behaviour of P. infestans 88069 and P. capsici LT1534 after exposure to DMSO. Both Phytophthora strains were exposed to 1.5% and 2.36% (v/v) DMSO/Plich for 10 days. DMSO levels correspond to the maximum capsidiol solution that was used in each experiment. The experiment was performed 3 times. (TIF) [file pone.0107462.s002.tif]
